# Recalcitrant Gastric Actinomycosis Treated With Over-the-Scope Clip

**DOI:** 10.14309/crj.0000000000000798

**Published:** 2022-06-23

**Authors:** Nicholas M. McDonald, Leticia P. Luz, Khalid Amin, Stuart K. Amateau

**Affiliations:** 1 Division of Gastroenterology, Hepatology, and Nutrition, University of Minnesota, Minneapolis, MN; 2 Division of Gastroenterology, HealthPartners Park Nicollet, St. Louis Park, MN; 3 Department of Laboratory Medicine and Pathology, University of Minnesota, Minneapolis, MN

## Abstract

Actinomycosis is an infrequent infection caused by *Actinomyces* species bacteria. Gastric actinomycosis is extremely rare but has been identified on endoscopy, typically presenting as erythema or ulceration. Standard therapies include prolonged antibiotics, and when these fail, gastric actinomycosis often requires surgical resection. We present a case of recalcitrant gastric actinomycosis, which presented as a subepithelial lesion and the first demonstration of treatment with endoscopic resection through over-the-scope clip.

## INTRODUCTION

Actinomycosis is a rare infection caused by Gram-positive *Actinomyces* species bacteria.^1,2^ Because of a low virulence, it is uncommon for actinomycosis to occur in an immunocompetent host, instead *Actinomyces* species commonly prey on those with immunocompromised condition or disrupted mucosa from surgical procedures.^3–6^ Actinomycosis can present with a variety of symptoms and findings based on the area involved and in some scenarios mimic malignancy.^2^ The most common locations for actinomycosis include the cervicofacial region, abdominopelvic region, and the respiratory tract.^1^ Gastric actinomycosis is rare and generally limited to case reports.^7,8^ In 1 series of 15 patients with actinomycosis, only 1 patient had gastric involvement.^9^ Detection during endoscopy is even more infrequent.^7,8,10^ We present a case of actinomycosis, which was found during endoscopy, and to the best of our knowledge, the first case to be treated with an over-the-scope clip (OTSC).

## CASE REPORT

**Figure 1.**
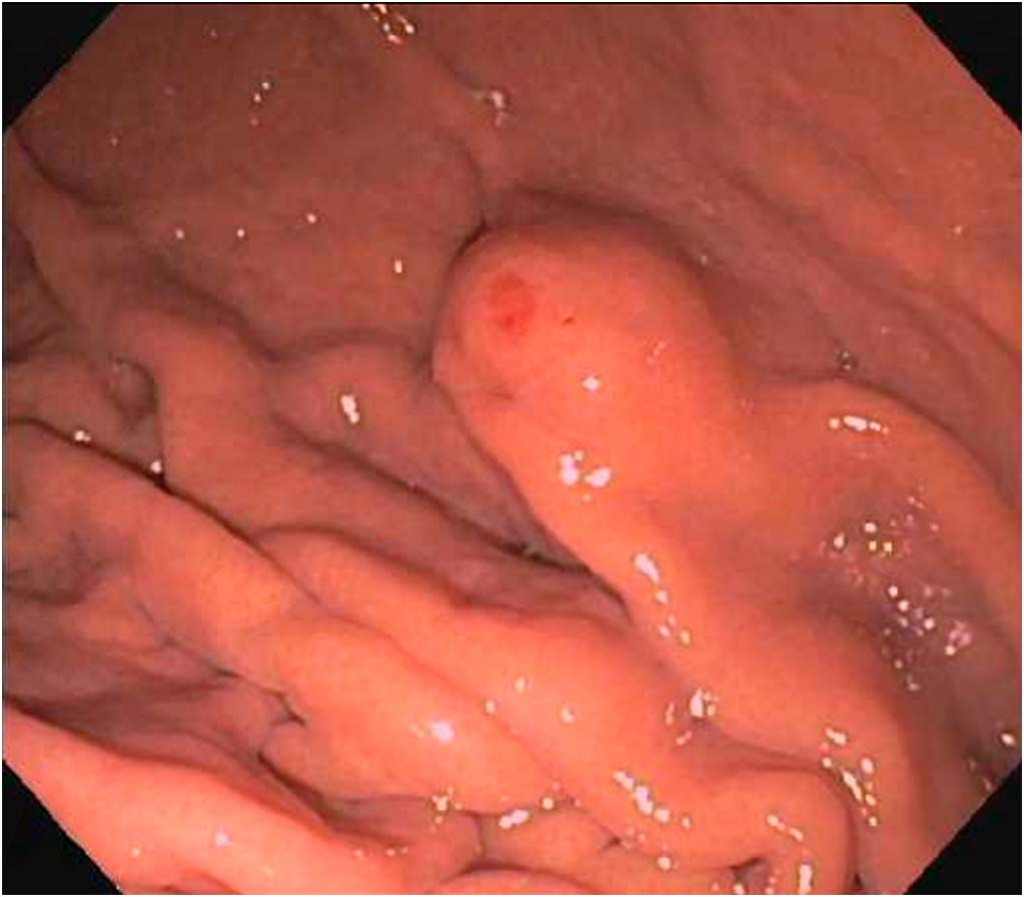
Gastric subepithelial lesion identified on esophagogastroduodenoscopy.

**Figure 2.**
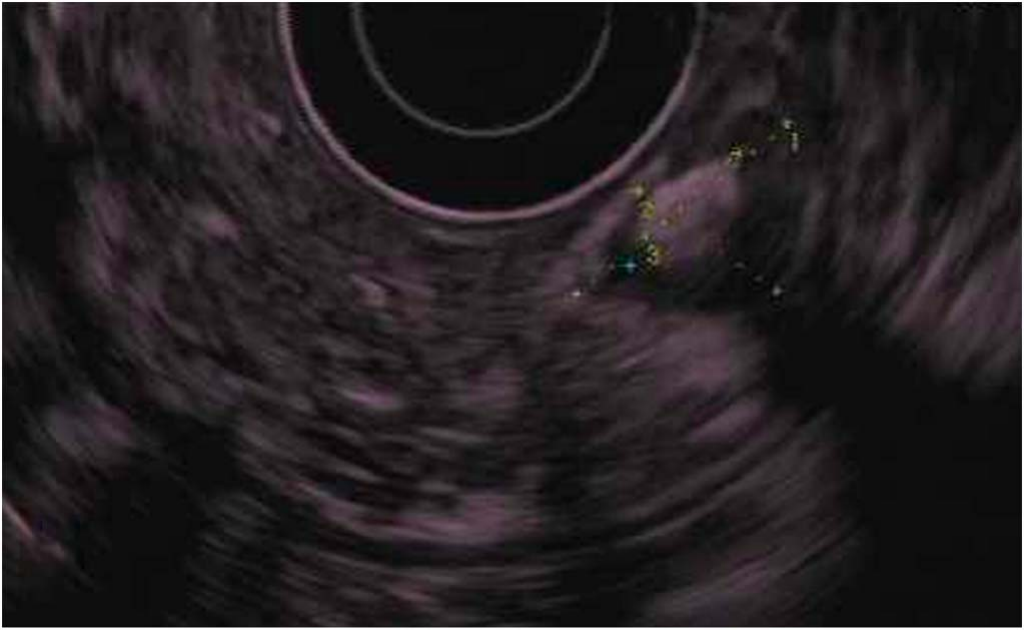
A 15-mm-by-10-mm hypoechoic and calcified gastric subepithelial lesion arising from the submucosa (layer 3), muscularis propria (layer 4), and intramural wall.

**Figure 3.**
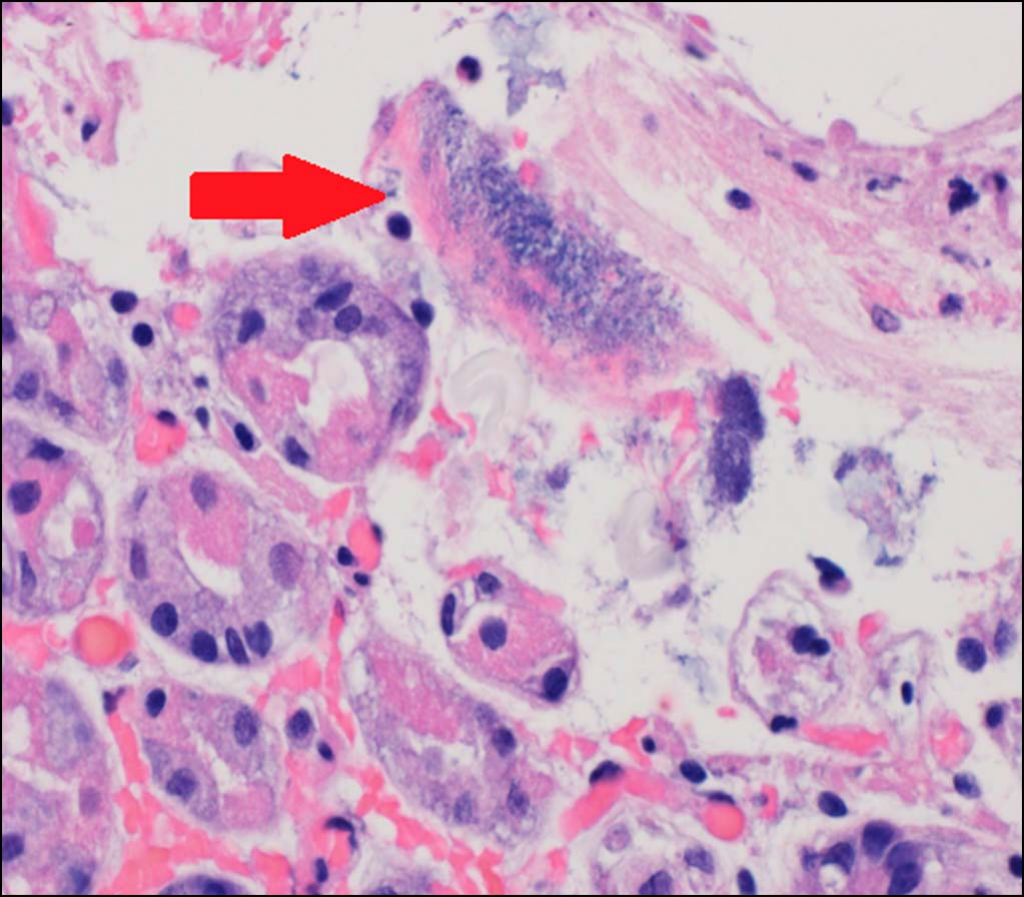
Pathology showing oxyntic gastric mucosa with filamentous *Actinomyces* organisms on the surface, which appear like cotton balls (red arrow).

**Figure 4.**
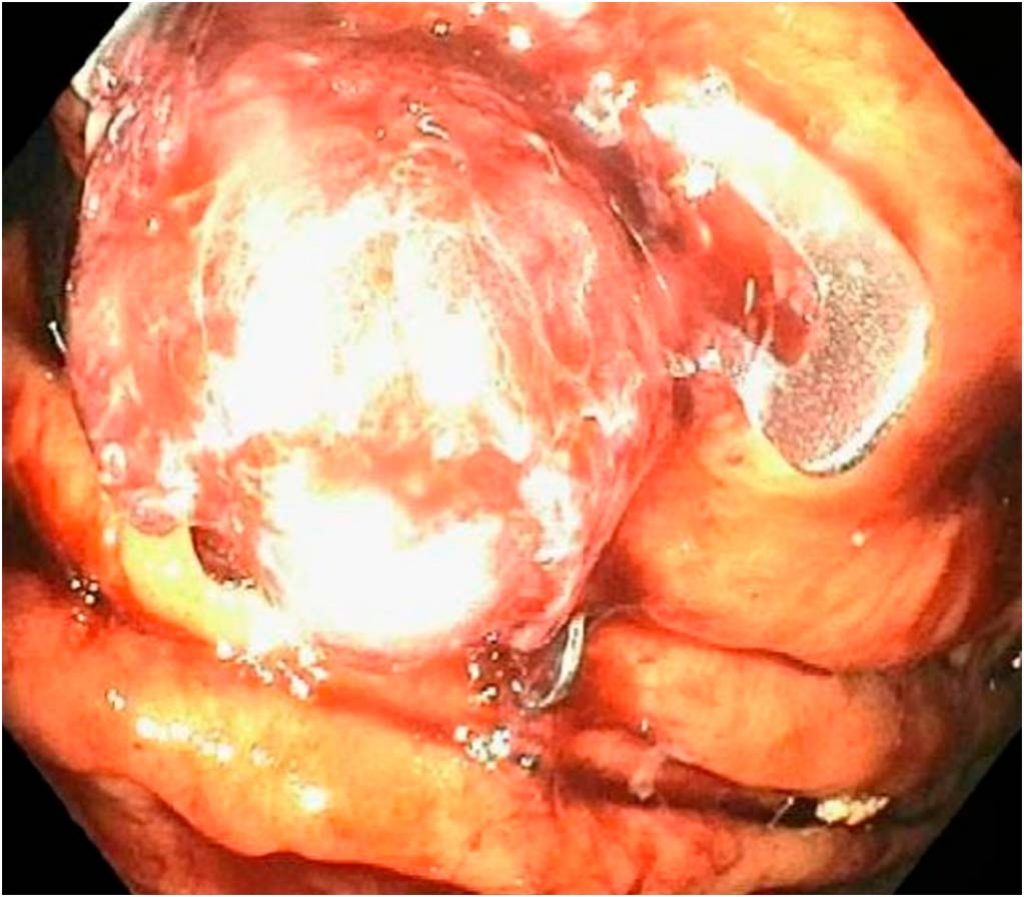
Final endoscopic view showing over-the-scope-clip deployment over the subepithelial lesion.

**Figure 5.**
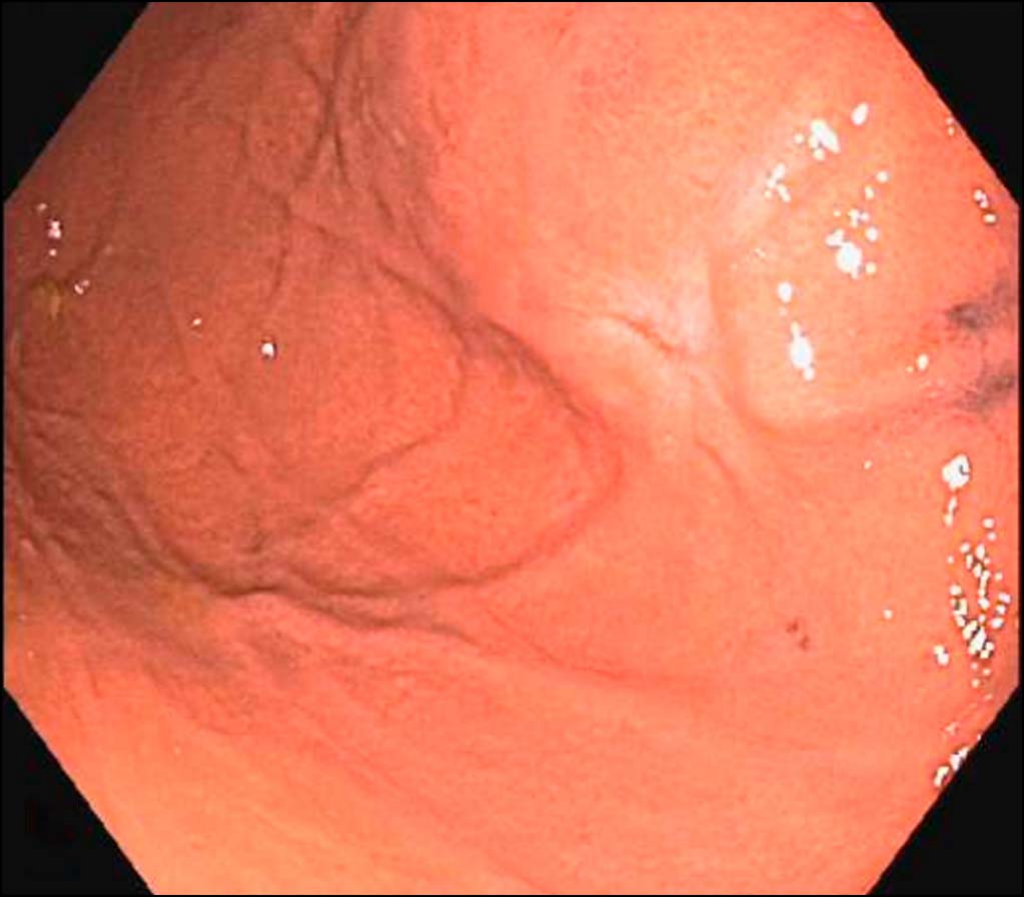
Upper endoscopy 3 months later showing the healed area.

A 70-year-old man with a medical history of hypertension presented for evaluation of heartburn and suspected gastroesophageal reflux disease. He underwent esophagogastroduodenoscopy, which demonstrated a subepithelial lesion in the gastric fundus (Figure 1). Subsequent endoscopic ultrasound demonstrated a 15-mm-by-10-mm hypoechoic and calcified lesion with shadowing arising from the submucosa (layer 3), muscularis propria (layer 4), and intramural wall (Figure 2). The lesion was sampled by fine-needle aspiration for cytologic interpretation as well as pathology, which revealed oxyntic gastric mucosa with Gram-positive filamentous bacteria, which appeared like cotton balls, consistent with *Actinomyces* (Figure 3). Cultures returned positive for *Actinomyces odontolyticus.* The patient was evaluated by infectious diseases and treated with intravenous penicillin, followed by a prolonged course of oral penicillin over the next 3 months. Despite antibiotic therapy, the lesion persisted, and the infectious disease team felt all medical options had been exhausted. Given his recalcitrant disease, the decision was made to proceed with resection, and based on shared decision-making, he was referred for consideration of endoscopic full-thickness resection (EFTR). During the procedure, the lesion was identified and targeted for EFTR; however, several devices were unsuccessful in grasping and pulling the lesion into the cap. This was believed to be due to the subepithelial lesion containing a firm, calcified core with soft overlying tissues. Because the lesion could not be pulled fully into the cap, the decision was made to deploy the OTSC alone without subsequent snare resection. After deployment, the lesion appeared above the clip, and the intention was to allow the lesion to slough off as the area necrosed (Figure 4). The patient did well after the procedure, and at the time of last follow-up 3 months later, the lesion was absent, and cultures from biopsies of the area had no further growth of *Actinomyces* (Figure 5).

## DISCUSSION

Actinomycosis is a rare condition caused by clinical infection from *Actinomyces* species.^1^ *Actinomyces* are opportunistic and are more likely to cause infection in an immunocompromised host or cases where the mucosal barrier is disrupted, allowing for direct invasion. Al-Obaidy et al^10^ previously published a case of actinomycosis, which was diagnosed through esophagogastroduodenoscopy with gastric biopsy. In their case, the patient had presented with abdominal pain, and suspected gastric outlet obstruction and endoscopically seemed to have gastric erythema.^10^ By contrast, Patel et al^8^ reported a case of gastric actinomycosis, which presented with hematemesis and an ulcer along the greater curvature. In the case reported by Patel et al,^8^ because of suspicion for malignancy and a lack of response to an extended course of penicillin, the patient underwent surgical resection of the involved area, and final pathology confirmed the diagnosis of gastric actinomycosis. A third case was reported by Lee at al,^11^ which was diagnosed on gastric biopsy. The diagnosis in this case was surprising, as generally *Actinomyces* is a disease of immunocompromised hosts, but in this case the patient as immunocompetent with only a history of hypertension. In comparison with previous reports, this case demonstrates the actinomycosis presenting as a calcified gastric subepithelial lesion, which possibly insulated the bacteria from antibiotics. Because of a lack of response to therapy and in collaboration with infectious diseases, the decision was made to perform EFTR of the involved area. Surgery was considered; however, the patient declined any surgical intervention, and thus, maximal endoscopic therapy was pursued. EFTR is a minimally invasive approach that allows for resection of the full or near full thickness of the gastric wall with simultaneous closure of the defect.^11,12^ Moreover, EFTR is commonly used for gastric subepithelial lesions as a less-invasive alternative to surgical resection.^11–13^ With EFTR, it is imperative to pull the tissue into the cap before deployment of the OTSC and resection. Because of an inability to pull the tissue into the cap, we chose to simply deploy the OTSC beyond the lesion and to not perform a full-thickness resection. Although the potential spread of the infection should be considered because of disruption of the mucosal barrier, in other cases of recalcitrant actinomycosis, surgery was pursued, which would also result in the disruption of the mucosal barrier, and there was no evidence of such dissemination.^8^ The aim was to endoscopically resect the entire area with suspected actinomycosis, and to the best of our knowledge, this is the first case successfully treated using this approach. Although resection should only be considered when standard therapies fail, we have shown that OTSC or potentially EFTR may be viable alternatives to surgical resection for gastric actinomycosis.^14^

## DISCLOSURES

Author contributions: N. McDonald, L. Luz, K. Amin, and S. Amateau wrote the manuscript. S. Amateau and L. Luz performed the procedures. All authors performed critical review and editing of the manuscript. S. Amateau is the article guarantor.

Financial disclosure: S. Amateau reports a financial relationship with Merit Endoscopy, Boston Scientific, US Endoscopy/Steris, Heraeus (consultant), Olympus (consultant and advisor), and Cook Medical (consultant and research support). All other authors have no financial disclosures to report.

Informed consent was obtained for this case report.
